# Familial Hypercholesterolemia (FH) in Iran: Findings from the Four-Year FH Registry

**DOI:** 10.1155/2021/9913969

**Published:** 2021-06-11

**Authors:** Golnaz Vaseghi, Marzieh Taheri, Kiyan Heshmat-Ghahdarijani, Mohammad Rayati, Sonia Zarfeshani, Ali Pourmoghaddas, Alireza Khosravi, Ehsan Zarepour, Parsa Keshavarzrad, Sina Arabi, Mohammadreza Azizi, Shaghayegh Haghjooy Javanmard, Jamshid Najafian, Nizal Sarrafzadegan

**Affiliations:** ^1^Applied Physiology Research Center, Cardiovascular Research Institute, Isfahan University of Medical Sciences, Isfahan, Iran; ^2^Interventional Cardiology Research Center, Cardiovascular Research Institute, Isfahan University of Medical Sciences, Isfahan, Iran; ^3^Heart Failure Research Center, Cardiovascular Research Institute, Isfahan University of Medical Sciences, Isfahan, Iran; ^4^Isfahan Cardiovascular Research Center, Cardiovascular Research Institute, Isfahan University of Medical Sciences, Isfahan, Iran; ^5^Hypertension Research Center, Cardiovascular Research Institute, Isfahan University of Medical Sciences, Isfahan, Iran; ^6^Eritron Medical Laboratory, Sheilk Mofid St., Isfahan, Iran; ^7^School of Population & Public Health, University of British Columbia, Vancouver, BC, Canada

## Abstract

**Background:**

Familial hypercholesterolemia (FH) is a common autosomal dominant disease. Its diagnosis in Iran was uncommon. Iran registry of FH (IRFH) has been started from 2017 from Isfahan. In this study, we report the four-year FH registry.

**Methods:**

The Iran FH registry is an ongoing study which is followed by a dynamic cohort. It has been started from 2017. The patients are selected from laboratories due to high cholesterol level and who have history of premature cardiovascular disease. The Dutch Lipid Clinic Network (DLCN) criteria are used for the detection of FH. Cascade screening is performed for detection of first-degree relative of patients.

**Results:**

Among the 997 individuals included in this registry, they were 522 (mean age 51.41 ± 12.91 year), 141 (mean age 51.66 ± 8.3 year), and 129 (mean age 41 ± 16.5 year) patients from laboratories, premature cardiovascular disease, and relatives, respectively. In total, 263 patients were diagnosed with probable or definite FH, and others were in the possible group. Low-density lipoprotein cholesterol (LDL) level was 141.42 ± 45.27 mg/dl in the laboratory group and 54.9% of patients were on LLT treatment. In patients with premature cardiovascular disease and FH, the LDL level was 91.93 ± 32.58 and was on LLT treatment. The LDL concentration in the first relative of FH patients was 152.88 ± 70.77 and 45.7% of them are on LLT therapy.

**Conclusions:**

Most of FH patients were underdiagnosed and undertreated before their inclusion in the IRFH. Cascade screening helps in the improvement of diagnosis.

## 1. Background

Familial hypercholesterolemia (FH), the most frequent autosomal dominant disease, is caused by mutations in the LDL receptor (LDLR), Apo lipoprotein B (Apo B), or less frequently proprotein convertase Subtilisin/Kexin type 9 (PCSK9) genes [1]. FH is associated with a high level of low-density lipoprotein cholesterol (LDL-C) and the risk of developing premature atherosclerotic events. However, the progression and severity of cardiovascular disease (CVD) among FH patients are not predictable [2]. A mutation in one allele of the mentioned genes causes heterozygous FH (HeFH) phenotypes; however, the mutation of two alleles causes a more severe homozygous FH (HoFH) phenotype; in this case, the blood level of LDL-C is very high [3].

Timely and effective diagnosis and lowering the level of serum LDL-C improve the life expectancy and reduce the risk of developing premature CVD in FH [4] patients. Despite the importance of early diagnosis, due to the differences in prevalence, ethnicity, and polygenicity [5] of FH, no international consensus approach still exists. Around 20% of estimated FH prevalent cases are diagnosed in most countries, and many of them are aware of their disease often after the first ASCVD event [1]. So, developing national FH registry plays a crucial role in the diagnosis and management of FH patients. In previous years, many published guidelines and consensus papers have an emphasis on the early FH screening programs and initiating lipid-lowering treatment (LLT) for FH population [6].

We established the Iran Registry of Familial Hypercholesterolemia (IRFH) in 2017, which was started from Isfahan, in central Iran. The IRFH aims to improve FH detection in Iranian patients with hyperlipidemia alone or accompanied with premature CVD. The registry consequently gets the patients' relatives to be screened [7]. Before 2017, FH diagnosis was rare, and a small study showed that most of them did not achieve LDL-C goal [8]. This report describes the initial clinical characteristics of FH patients in Iran. Data has been obtained from IRFH.

## 2. Method

### 2.1. Study Population

FH registry (5) started in Isfahan from 2017 and is still ongoing. Ethical approval was obtained from the Isfahan University of Medical Sciences research ethics committee. The complete methodological approach has been explained elsewhere [7]. Briefly, the inclusion criteria were all subjects aged 2-80 years with LDL − C ≥ 190 mg/dl and not using lipid-lowering therapy (LLT) or corrected LDL − C ≥ 190 mg/dl [9] or patients with premature CVD (men less than 55 years and women less than 60 years old). The exclusion criteria were the secondary cause of hyperlipidemia and triglyceride levels > 400 mg/dl. To perform CASCADE screening, first-degree relatives of patients were invited. All the participants signed the consent forms.

### 2.2. Clinical and Laboratory Data Collection

Clinical examination was performed for evaluating the presence of tendon xanthoma, xanthelasma, or corneal arcus; they all asked about the previous history of premature CVD and family history of hyperlipidemia and premature CVD. All participants underwent a complete blood test consisting of high-density lipoprotein cholesterol (HDL-C), LDL-C, triglyceride (TG), fasting blood sugar (FBS), and total cholesterol (TC).

Diagnostic classification by clinical criteria was evaluated according to the Dutch Lipid Clinic Network (DLCN) criteria. In brief, people are divided into 4 categories: those with scores below 3 very unlikely have FH, scores above 8 are definitely affected by FH, scores between 6 and 8 probably suffer from FH, and scores between 3 and 5 possibly suffer from FH [10]. In cases of definite or probable FH, clinical diagnosis of FH (“clinical FH”) was made and CASCADE screening recommended in the first-degree relatives.

### 2.3. Statistical Analysis

Data were entered to EPI Info 7. For qualitative variables, data were shown as frequency (percentage), and for quantitative variables, data were shown as mean ± SD. All statistical analysis was performed using IBM SPSS statistics 25 software (IBM Corp. Released 2017. IBM SPSS Statistics for Windows, Version 25.0. Armonk, NY: IBM Corp.).

## 3. Results

Overall, 977 patients were registered in IRFH between 2017 and 2020. We present their results according to their recruitment type.

### 3.1. Characteristics of FH Patients Referred from Laboratories

The characteristics of 522 patients, who were screened according to their high LDL level from laboratories, are detailed in Table 1. The patients have been categorized by their Dutch score (66.9% with possible and 34.1% with definite/probable). The population was predominantly female (56.4%) with a mean age of 51 years. Overall, 29% had a family history of a cardiovascular event; however, it was 46.7% in the definite/probable group. Most of them never smoke (83%). The presence of Achilles tendon, xanthoma, and arcus cornealis was mostly observed in the definite/probable group (8.4%, 28%, and 4%, respectively). More than 27% have hypertension, while diabetes and history of CVD were observed in 17 and 18%, respectively.

LDL level was 141.42 ± 45.27 mg/dl; the highest LDL concentration was calculated in a definite group (152.09 ± 56.86 mg/dl). The concentration of total cholesterol was 234.94 ± 58.94 mg/dl, and TG concentration was 164.97 ± 69.1 mg/dl. The blood concentrations of HDL and non-HDL cholesterol were 47.69 ± 1.2 and 187.25 ± 56.84 mg/dl, respectively. 54.9% of patients were on LLT treatment, 19.5% were on antihypertensive treatment, and 11.9% of them took antidiabetic medication.

### 3.2. Characteristics of FH Patients with Premature Coronary Vascular Disease

Table 2 shows the baseline characteristics of FH patients with premature CVD. Among the 425 patients, 86.6% were classified as possible FH, and 13.2% were classified as definite/probable. Males constitute 63.1%, and mean age is 51.66 ± 8.31 years. In total, 41% had a family history of premature CVD. 68% of patients never smoke. The presence of Achilles tendon, xanthoma, and arcus cornealis in the definite/probable group was as follows: 16.1%, 28.6%, and 1.8%. In general, 63.5% had hypertension, and 28.2% had been diagnosed with diabetes. LDL-C concentration was 89.01 ± 27.32 mg/dl, 112.38 ± 34.43 mg/dl, and 110.47 ± 81.15 mg/dl in possible, probable, and definite group, respectively. Total cholesterol concentration was 168 ± 45.48 mg/dl. The blood concentration of HDL was 45.87 ± 11.56. As they all had history of premature CVD, most of them were on LLT (77.9%).

### 3.3. Characteristics of First Relatives with FH

Table 3 shows the result of cascade screening. Overall, 309 individuals were screened, and 129 were diagnosis with FH. The age of diagnosis in this group was 41 ± 16.5. Xanthomas was detected in 47.1% of patients in the definite group, and 23.5% of patients in this group had the presence of Achilles tendon. Overall, 28.9% of patients had a history of CVD. The LDL concentration in this group was 152.88 ± 70.77, and total cholesterol concentration was 243.51 ± 95.1. However, less than half of them were on LLT (45.7%).

## 4. Discussion

IRFH is the first registry with comprehensive approach which examined all characteristics of FH patients in Iran. Within three years, we were able to identify 977 patients with FH from laboratories, hospitals, and cascade screening. The overall LDL-C was about 141, 91, and 155 mg/dl in patients from laboratories, with premature CVD and from families of FH patients, respectively. Most of the patients were categorized in the possible group. Our study demonstrates that FH patients were poorly diagnosed and managed before registration in IRFH. The mean age was 51 for patients from laboratories and premature CVD at the registry entry, and it was ten years younger during family screening, which emphasizes on cascade screening [11].

It has been suggested that FH prevalence is between 1 : 150 and 1 : 250 in some populations [1, 12]. If it is assumed that FH prevalence in Iran is 1 : 250, and 2000000 population of Isfahan, it can be concluded that we identified 3% in 12% with all above Dutch score of more than 3.

Worldwide several existing FH registries include patients based on either clinical or genetic diagnosis or both [13]. However, despite that, many FH patients are still underdiagnosed in many countries [1]. Different screening methods are used to detect FH patients, for example, screening of the general population or screening patients who are admitted to acute coronary units with premature myocardial infarction or family cascade screening of patients with FH [14]. In Isfahan registry, we identified suspected FH cases through laboratories, hospital systems, and also, family screening. The majority of patients were identified as possible FH in all three groups, according to the DLCN criteria. This could be because of clinical presentation such as tendon xanthomas or family history missing which leads to missclassification of FH patients. This has been observed in other registries, too [15].

Tendon xanthomas were present in less than 10% of patients which is lower than several other reports [16]. The low prevalence of xanthomas can be explained because of including patients with possible Dutch criteria in our cohort. In this registry, we reported premature arcus cornealis (diagnosed before age 45). As many patients were older than 45 years, the prevalence of arcus cornealis was low.

We investigated the prevalence of other nonlipid risk factors in this study, such as active smoking, BMI, HTN, and diabetes. The prevalence of hypertension was higher in patients with premature CVD (63%) compared with two other groups (27% and 20 5 in patients from laboratories and cascade screening 1, respectively). It seems that HTN is higher in our registry than individuals from the Iranian adult cohort (5%). The prevalence of diabetes was higher in FH patients with premature CVD compared with two other groups [17]. The rates of overweight were as follows in our three groups: 27, 27, and 26 kg/m in patients from laboratories, premature CVD, and cascade screening. However, the slight reduction in the last group may be because of younger age.

The risk of developing ASCVD in FH is more than seven times compared with normolipidemic individuals [18]. Early diagnosis and treatment initiation in FH patients are associated with reduced incidence of premature CVD. Data indicate that when promptly treated with statins, FH patients do not differ from the general population regarding CVD incidence Statins are the first-line therapy for FH [19] patients. In Isfahan registry, only 54% of patients from laboratories and about 80% of FH patients with premature CVD were on LLT, as expected that was lower in patients who were diagnosed with FH during cascade screening.

There were some limitations in our study; first, we were not able to access to primary LDL-C concentration of some patients, and in some cases, statin therapy could diminish the xanthomas. Second, in some cases, we could not assess the family history of patients which might underscore some patients. Third, we used a phenotypic diagnosis, and we could not perform the genetic test in the majority of patients; however, in our previous study, we showed the *APOB* mutation in our sample [20].

One of the most important messages from our study is FH registry should be expanded in other Iran cities. These local registries should aware general physicians and cardiologists to identify new cases. Our study revealed that FH relatives has a younger age than FH cases, similar to other studies, so early detection and cascade screening are the most important factors in the management of FH [1, 21].

In conclusion, our results showed that many FH patients are underdiagnosed and undertreated; FH in Greece is characterized by a significant delay in the diagnosis and a high prevalence of both family and personal history of established CVD. The vast majority of FH patients do not achieve LDL-C targets. Improved awareness and management of FH are definitely needed. Plans need to develop tools to support practice physicians to conduct clinics to ensure that the assessment and management of FH patients is ongoing. The FH cohort study has been started in Iran, and lipid level can be monitored [22].

## Figures and Tables

**Table 1:** Baseline characteristics of subjects with familial hypercholesterolemia by Dutch Lipid Clinic Network diagnosis.

	Possible (*n* = 350 (66.9%)	Probable (*n* = 98 (18.7%))	Definite (*n* = 75 (14.3%))	Definite and probable (*n* = 173 (33.1%))	Total (*n* = 523)
Demographics					
Sex (male)	169 (48.4)	37 (37.8)	27 (36.0)	64 (37.0)	233 (44.6)
Age (years)	51.13 ± 12.72	51.33 ± 13.95	52.84 ± 12.46	51.98 ± 13.31	51.41 ± 12.91
Family history of premature CVD^a^	72 (21.0)	51 (54.3)	26 (36.6)	77 (46.7)	149 (29.3)
Smoking status					
Never	292 (83.4)	77 (78.6)	68 (90.7)	145 (83.8)	437 (83.6)
Current	42 (12)	15 (15.3)	3 (4)	18 (10.4)	60 (11.5)
Previous	16 (4.6)	6 (6.1)	4 (5.3)	10 (5.8)	26 (50.0)
Presence of Achilles tendon	0 (0)	1 (1.1)	13 (18.3)	14 (8.4)	14 (2.8)
Xanthomas	0 (0)	7 (7.3)	42 (56.8)	49 (28.8)	49 (9.6)
Arcus cornealis	0 (0)	6 (6.1)	1 (1.3)	7 (4.0)	7 (1.3)
Body mass index (kg/m^2^)	27.62 ± 4.49	27.45 ± 3.94	27.56 ± 5.01	27.5 ± 4.38	27.58 ± 4.45
Comorbidities					
Hypertension	82 (23.6)	38 (40.4)	20 (27.4)	58 (34.7)	140 (27.2)
Diabetes mellitus	56 (16.3)	20 (22.2)	14 (19.7)	34 (21.1)	90 (17.8)
History of CVD	33 (9.4)	46 (46.9)	17 (22.7)	63 (36.4)	96 (18.4)
Biochemical measures					
Total cholesterol (mg/dl)	229.98 ± 56.94	244.1 ± 59.91	246.0 ± 64.44	244.92 ± 61.7	234.94 ± 58.94
LDL-cholesterol^b^ (mgr/dl)	137.27 ± 42.49	148.06 ± 43.21	152.09 ± 56.86	149.81 ± 49.48	141.42 ± 45.27
HDL-cholesterol (mgr/dl)	47.7 ± 11.81	47.96 ± 10.53	47.27 ± 9.6	47.66 ± 10.11	47.69 ± 11.26
Non-HDL cholesterol (mg/dl)	182.27 ± 54.9	196.14 ± 56.69	198.73 ± 63.3	197.27 ± 59.48	187.25 ± 56.84
Triglycerides (mgr/dl)	166.24 ± 69.59	160.3 ± 70.2	165.05 ± 65.95	162.37 ± 68.22	164.97 ± 69.1
FBS^d^	100.08 ± 29.37	101.39 ± 34.76	95.32 ± 14.90	98.76 ± 28.04	99.64 ± 28.91
HbA1c^e^	5.82 ± 1.35	5.86 ± 1.20	5.67 ± 0.75	5.77 ± 1.03	5.81 ± 1.25
History of pharmacotherapy					
Lipid-lowering drugs	168 (48)	65 (66.3)	54 (72)	119 (68.8)	287 (54.9)
Antihypertensive	65 (18.6)	25 (25.5)	12 (16)	37 (21.4)	102 (19.5)
Antidiabetics	38 (10.9)	16 (16.3)	8 (10.7)	24 (13.9)	62 (11.9)
Aspirin	31 (8.9)	13 (13.3)	6 (8.0)	19 (11.0)	50 (9.6)

Data are shown as mean ± SD or frequency (percentage). ^a^CVD: cardiovascular disease. ^b^LDL-cholesterol: low-density lipoprotein cholesterol. ^c^HDL-cholesterol: high-density lipoprotein cholesterol. ^d^FBS: fast blood sugar. ^e^HbA1c: hemoglobin A1c.

**Table 2:** Baseline characteristics of patients with premature coronary heart disease and familial hypercholesterolemia, by Dutch Lipid Clinic Network diagnosis.

	Possible (*n* = 369 (86.8%))	Probable (*n* = 37 (8.7%))	Definite (*n* = 19 (4.5%))	Definite and probable (*n* = 56 (13.2%))	Total (*n* = 425)
Demographics					
Sex (male)	238 (64.5)	22 (59.5)	8 (42.1)	30 (53.6)	268 (63.1)
Age (years)	51.49 ± 8.03	53.95 ± 7.59	50.53 ± 13.31	52.79 ± 9.92	51.66 ± 8.31
Family history of premature CVD^a^	145 (39.4)	19 (51.4)	11 (57.9)	30 (53.6)	175 (41.3)
Smoking status					
Never	248 (67.2)	30 (81.1)	12 (63.2)	42 (75)	290 (68.2)
Current	82 (22.2)	3 (8.1)	3 (15.8)	6 (10.7)	88 (20.7)
Previous	39 (10.6)	4 (10.8)	4 (21.1)	8 (14.3)	47 (11.1)
Presence of Achilles tendon	0 (0)	3 (8.1)	6 (31.6)	9 (16.1)	9 (2.1)
Xanthomas	0 (0)	3 (8.1)	13 (68.4)	16 (28.6)	16 (3.8)
Arcus cornealis	0 (0)	1 (2.7)	0 (0)	1 (1.8)	1 (0.2)
Body mass index (kg/m^2^)	28.06 ± 4.44	29.21 ± 3.81	27.93 ± 4.02	28.78 ± 3.89	28.16 ± 4.37
Comorbidities					
Hypertension	242 (65.6)	23 (62.2)	5 (26.3)	28 (50)	270 (63.5)
Diabetes mellitus	101 (27.4)	13 (35.1)	6 (31.6)	19 (33.9)	120 (28.2)
History of CVD	369 (100)	37 (100)	19 (100)	56 (100)	425 (100.0)
Biochemical measures					
Total cholesterol (mg/dl)	163.42 ± 39.19	204.59 ± 49.44	187.29 ± 98.43	199.15 ± 68.19	168.0 ± 45.48
LDL-cholesterol^b^ (mgr/dl)	89.01 ± 27.32	112.38 ± 34.43	110.47 ± 81.15	111.78 ± 52.86	91.93 ± 32.58
HDL-cholesterol^c^ (mgr/dl)	45.51 ± 11.07	47.73 ± 10.46	49.44 ± 20.38	48.29 ± 14.29	45.87 ± 11.56
Non-HDL cholesterol (mg/dl)	117.91 ± 35.95	156.86 ± 45.36	141.94 ± 94.72	152.17 ± 64.46	122.31 ± 42.22
Triglycerides (mgr/dl)	152.39 ± 65.63	197.68 ± 75.17	120.61 ± 39.12	172.45 ± 74.70	155.02 ± 67.14
FBS^d^	114.59 ± 48.33	126.32 ± 46.81	122.78 ± 62.69	125.16 ± 51.97	115.97 ± 48.89
HbA1c^e^	5.88 ± 1.96	6.54 ± 2.19	6.53 ± 1.55	6.54 ± 2.01	5.97 ± 1.98
History of medication use					
Lipid-lowering drugs	281 (76.2)	35 (94.6)	15 (78.9)	50 (89.3)	331 (77.9)
Antihypertensive	269 (72.9)	30 (81.1)	8 (42.1)	38 (67.9)	307 (72.2)
Antidiabetics	98 (26.6)	14 (37.8)	5 (26.3)	19 (33.9)	117 (27.5)
Aspirin	242 (65.6)	30 (81.11)	11 (57.9)	41 (73.2)	283 (66.6)

Data are shown as mean ± SD or frequency (percentage). ^a^CVD: cardiovascular disease. ^b^LDL-cholesterol: low-density lipoprotein cholesterol. ^c^HDL-cholesterol: high-density lipoprotein cholesterol. ^d^FBS: fast blood sugar. ^e^HbA1c: hemoglobin A1c.

**Table 3:** Baseline characteristics of subjects with familial hypercholesterolemia by cascade screening.

	Possible (*n* = 95 (73.6%))	Probable (*n* = 16 (12.4%))	Definite (*n* = 18 (14.0%))	Definite and probable (*n* = 34 (26.4%))	Total (*n* = 129)
Demographics					
Sex (male)	46 (48.4)	8 (50.0)	8 (44.4)	16 (47.1)	62 (48.1)
Age (years)	42.12 ± 16.33	41.25 ± 17.59	35.56 ± 16.15	38.24 ± 16.83	41.0 ± 16.5
Family history of premature CVD^a^	39 (41.1)	7 (43.8)	3 (16.7)	10 (29.4)	49 (38.0)
Smoking status					
Never	80 (84.2)	13 (81.3)	15 (83.3)	28 (82.4)	108 (83.7)
Current	9 (9.5)	3 (18.8)	2 (11.1)	5 (14.7)	14 (10.9)
Previous	6 (6.3)	0 (0.0)	1 (5.6)	1 (2.9)	7 (5.4)
Presence of Achille tendon	0 (0)	0 (0)	4 (23.5)	4 (12.1)	4 (3.1)
Xanthomas	0 (0)	0 (0)	8 (47.1)	8 (24.2)	8 (6.3)
Arcus cornealis	0 (0)	1 (6.3)	2 (11.1)	3 (8.8)	3 (2.3)
Body mass index (kg/m^2^)	26.96 ± 5.58	24.75 ± 3.94	25.63 ± 4.41	25.23 ± 4.16	26.52 ± 5.29
Comorbidities					
Hypertension	24 (25.3)	2 (12.5)	1 (5.6)	3 (8.8)	27 (20.9)
Diabetes mellitus	13 (13.8)	2 (12.5)	5 (27.8)	7 (20.6)	20 (15.6)
History of CVD	31 (33.0)	1 (6.3)	5 (27.8)	6 (17.6)	37 (28.9)
Biochemical measures					
Total cholesterol (mg/dl)	221.91 ± 56.98	248.25 ± 85.53	352.06 ± 168.28	303.21 ± 143.80	243.51 ± 95.13
LDL-cholesterol^b^ (mgr/dl)	136.98 ± 44.35	163.88 ± 69.23	226.17 ± 123.41	196.85 ± 104.97	152.88 ± 70.77
HDL-cholesterol^c^ (mgr/dl)	49.03 ± 12.06	48.19 ± 12.34	52.78 ± 19.22	50.62 ± 16.27	49.45 ± 13.25
Non-HDL cholesterol (mg/dl)	173.09-55.31	200.06 ± 78.07	299.28 ± 154.32	252.59 ± 132.54	194.20 ± 89.71
Triglycerides (mgr/dl)	141.39 ± 72.79	140.88 ± 68.89	170.11 ± 138.69	156.35 ± 110.84	145.37 ± 84.36
FBS^d^	103.99 ± 50.93	95.00 ± 21.37	94.72 ± 15.63	94.85 ± 18.26	101.52 ± 44.64
HbA1c^e^	5.38 ± 1.49	5.71 ± 0.86	5.38 ± 0.89	5.54 ± 0.88	5.42 ± 1.36
History of medication use					
Lipid-lowering drugs	38 (40.0)	9 (56.3)	12 (66.7)	21 (61.8)	59 (45.7)
Antihypertensive	22 (23.2)	3 (18.8)	1 (5.6)	4 (11.8)	26 (20.2)
Antidiabetics	8 (8.4)	1 (6.3)	2 (11.1)	3 (8.8)	11 (8.5)
Aspirin	12 (12.6)	2 (12.5)	1 (5.6)	3 (8.8)	15 (11.6)

^a^CVD: cardiovascular disease. ^b^LDL-cholesterol: low-density lipoprotein cholesterol. ^c^HDL-cholesterol: high-density lipoprotein cholesterol. ^d^FBS: fast blood sugar. ^e^HbA1c: hemoglobin A1c.

## Data Availability

The collection of data that supports the findings in this study is available from the Isfahan Cardiovascular Research Institute of Isfahan University of Medical Sciences in Isfahan Iran, but restrictions may apply to the availability of these data, which were used under license for the current study, and so are not publicly available. Data are however available from the authors upon reasonable request and with permission of Isfahan Cardiovascular Research Institute.
